# Distribution profiles of diphenhydramine and lidocaine in scalp, axillary, and pubic hairs measured by micro-segmental hair analysis: good indicator for discrimination between administration and external contamination of the drugs

**DOI:** 10.1007/s11419-021-00590-x

**Published:** 2021-07-31

**Authors:** Kenji Kuwayama, Hajime Miyaguchi, Tatsuyuki Kanamori, Kenji Tsujikawa, Tadashi Yamamuro, Hiroki Segawa, Yuki Okada, Yuko T. Iwata

**Affiliations:** National Research Institute of Police Science, 6-3-1 Kashiwanoha, Kashiwa, Chiba 277-0882 Japan

**Keywords:** Micro-segmental hair analysis, Administration or external contamination, Diphenhydramine, Lidocaine, Axillary and pubic hairs

## Abstract

**Purpose:**

Drug distribution in scalp hair can provide historical information about drug use, such as the date and frequency of drug ingestion. We previously developed micro-segmental hair analysis, which visualizes drug distribution at 0.4-mm intervals in individual hairs. The present study examines whether the distribution profiles of drugs can be markers for the administration or external contamination of the drugs using scalp, axillary, and pubic hairs.

**Methods:**

A single dose of anti-itch ointment containing diphenhydramine (DP) and lidocaine (LD) was topically applied to the axillary or pubic areas of two volunteers; DP was also orally administered; and LD was intra-gingivally injected. Scalp, axillary, and pubic hairs were assessed using our micro-segmental analysis.

**Results:**

The localization of DP and LD differed within individual scalp hair strands, implying DP and LD were predominantly incorporated into scalp hair via the bloodstream and via sweat/sebum, respectively, showing double-peak profiles. However, DP and LD were distributed along the shafts of axillary and pubic hairs without appearance of the double-peak profiles when the ointment had been applied to the axillary and pubic areas. The distributions of DP and LD in scalp hairs did not significantly differ according to administration routes, such as oral administration, gingival injection, and topical application.

**Conclusions:**

Micro-segmental analysis revealed differences in the distribution profiles of drugs in hairs, and distinguished hairs with and without external contamination. These findings will be useful for understanding of the mechanism of drug uptake into hair and for estimating the circumstances for a drug use.

**Supplementary Information:**

The online version contains supplementary material available at 10.1007/s11419-021-00590-x.

## Introduction

Understanding how a drug is administered is important to elucidate the circumstances of drug-related crimes during investigations. Drugs can be administered via various routes, such as orally, topically, intravenously and by inhalation [1–5]. The choice of administration route affects the time course of drug concentrations in the blood and can change the duration of pharmacological effects. Drug abusers sometimes claim in court that drugs detected in their biological specimens are not from drugs that they deliberately ingested but from accidental physical contact with drugs.

**Fig. 1:**
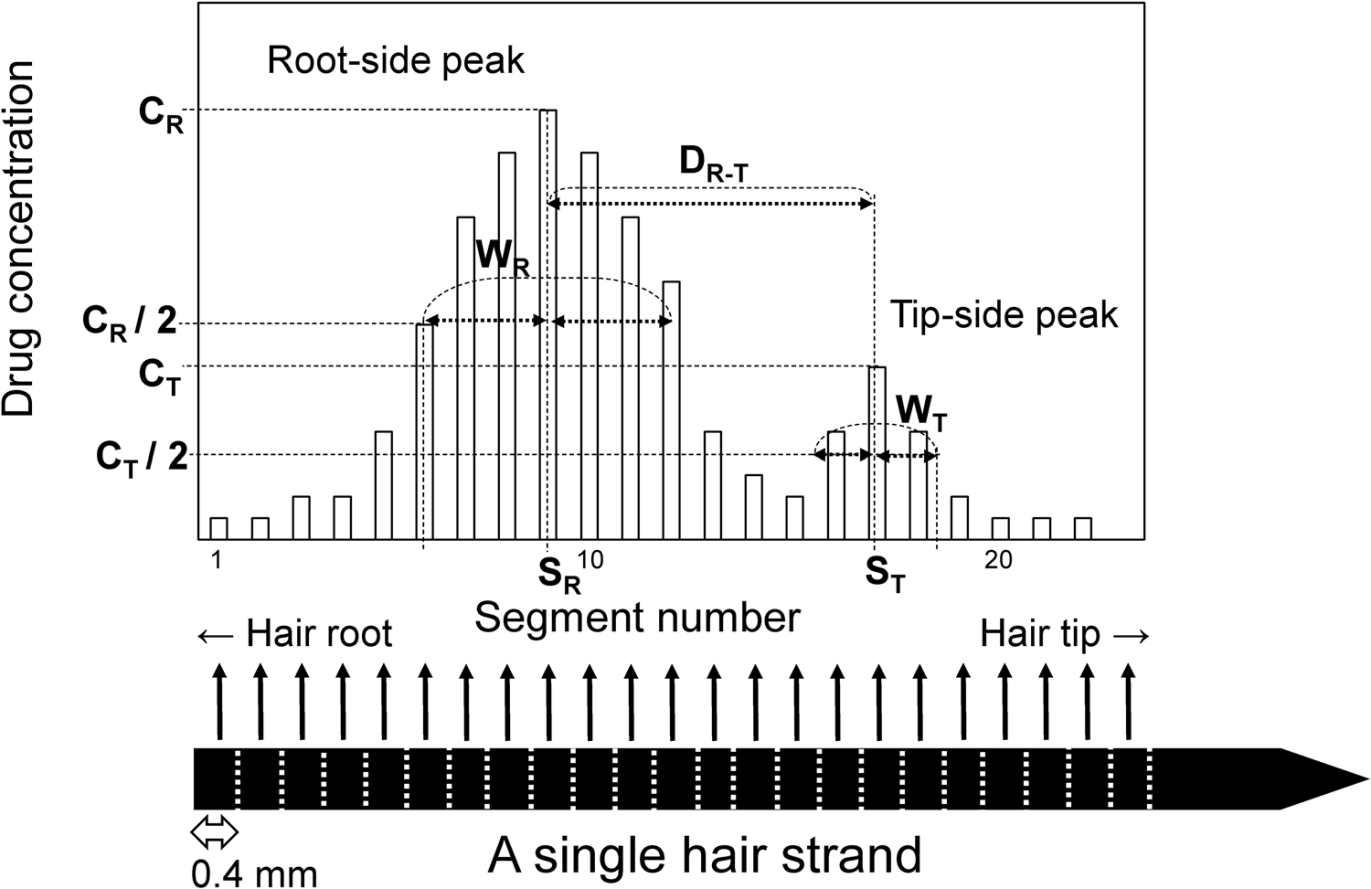
Typical drug distribution profile for single hair strand and peak parameters

One method of estimating routes of drug administration is to monitor blood drug concentrations [6, 7]. However, this requires obtaining many blood samples for several days soon after drug administration. This is not practical in criminal investigations because biological specimens from suspects or victims are usually collected only once after an incident is brought to the attention of police, which can be a long time after the drug ingestion. Hair also has a potential to estimate drug administration routes because information about the time course of drug concentration may be recorded in individual hair strands for several months or more [8–10]. However, conventional analyses of hair segments of several centimeters cannot determine differences in drug distribution profiles among administration routes, because details cannot be obtained. We previously developed micro-segmental hair analysis, which visualizes drug distribution in detail by segmenting a single hair strand at 0.4-mm lengths [11]. In micro-segmental analysis, a two-hump shaped profile with two peaks as shown in Fig. 1 is typically obtained when a single dose of drug is orally ingested. It is considered that the root-side and tip-side peaks are due to the uptake of drugs from bloodstream in the hair follicle, and from sweat and sebum secreted by sweat and sebaceous glands near the scalp, respectively [12–14]. If the peak shape changes according to the administration route, the analytical results would provide valuable information about how a drug was administered during drug-related crimes.

In this study, we compared the profiles of the model drugs, diphenhydramine (DP) and lidocaine (LD) in hair administered via different routes. Both model drugs are ingredients in over-the-counter (OTC) medicines. DP is sold in the tablet form to improve sleep quality and as an anti-itching ointment [15]. LD is often mixed with DP in anti-itching ointment [16], and it is administered as a local anesthetic by physicians for procedures such as endoscopy [17], and by dentists [18]. Differences in the profiles of topically applied DP and LD, orally administered DP and gingivally injected LD in scalp hair were examined using the micro-segmental analysis.

We also compared the profiles of DP and LD in hair from the scalp, axillary, and pubic area; the ointment was topically applied to the axillary or pubic areas. Although drugs have been tested in axillary and pubic hairs [19–22], their individual hair strands have not yet been analyzed to our knowledge. The effectiveness of axillary and pubic hairs as alternative specimens to scalp hair in micro-segmental analysis, and the distinction between hairs contaminated with drugs and hairs after drug administration were also discussed herein.

## Materials and methods

### Materials

OTC pharmaceutical products, Feminina^®^ (an anti-itch ointment containing DP and LD), Wutt^®^ (a sedative-hypnotic medicine containing DP), and Stac^®^ (a common cold medicine containing chlorpheniramine (CP) and methylephedrine (ME)) were purchased from Kobayashi Pharmaceutical Co., Ltd. (Osaka, Japan), Itami Pharmaceutical Co., Ltd. (Takashima, Japan), and SSP Co., Ltd. (Tokyo, Japan), respectively. Reference standards, LD and desethyllidocaine (DLD) were purchased from Sigma-Aldrich Co. LLC (St. Louis, MO, USA). CP-*d*_6_ maleate was purchased from Santa Cruz Biotechnology, Inc. (Dallas, TX, USA). DP hydrochloride, desmethyldiphenhydramine (DDP) hydrochloride, and the other reagents were purchased from FUJIFILM Wako Pure Chemical Corporation (Osaka, Japan). Acetonitrile, methanol, and water were of liquid chromatography/mass spectrometry grade.

### Drug administration

Participant A was an Asian man aged in his 40s with black scalp hair, and participant B was an Asian woman in her 30s with black scalp hair that was dyed brown. Both participants applied 1.0 g of Feminina^®^ ointment containing DP hydrochloride (20 mg) and LD (20 mg) to their axillary or pubic areas. The skin and hair in/around the areas where the ointment was applied were washed about 24 h later. One Wutt^®^ tablet containing DP hydrochloride (8.3 mg) was orally administered as a single dose to both participants. Both participants had ingested Stac^®^ common cold medicine containing CP (2.5 mg) and ME (20 mg) as a single dose with intervals of over 2 weeks as internal temporal markers, which marks a timescale in a hair strand [23, 24]. Both had received gingival injection of LD (6–36 mg) for local anesthesia during dental treatments.

A few strands of scalp hair were plucked from the posterior vertex regions of the heads of the participants several weeks after drug administrations. A few strands of axillary and pubic hairs were plucked at the same time as scalp hair. DP and LD were topically applied, orally administered and injected with intervals of at least 1 month. The participants affirmed that they had not taken any other medicines containing DP, LD, CP, and ME during the study.

### Micro-segmental analysis

The detailed sample preparation is described in our previous reports [11, 12]. Briefly, an individual hair strand was weighed and its full length was measured. After each hair strand was washed by sonicating with 1% sodium dodecyl sulfate (SDS) aqueous solution (1.5 mL) for 1 min, it was washed alternately with water (1.5 mL) and methanol (1.5 mL) 3 times for 1 min each. The hair strand was cut in a 0.4-mm length from the end of the hair root side using a tissue slicer equipped with a micrometer scale (Stoelting Co., Wood Dale, IL, USA), and each segment was placed in a 0.1-mL microtube. A mixture of the mobile phase consisting of an aqueous solution of 5 mM ammonium acetate plus 0.05% formic acid (mobile phase A) and acetonitrile (3:1, by vol.) was used as the extraction solution. Then, the extraction solution (100 μL) containing CP-*d*_6_ (4 pg/mL) as the internal standard was added to the tube containing each 0.4-mm segment. The sample tube was then sonicated at 23 kHz for 10 min and then maintained at approximately 22 °C in a dark place for 24 h. The supernatant (35 μL) was transferred to a 96-well plate and then diluted with mobile phase A (35 μL), and the resultant solution (50 μL) was injected into a liquid chromatograph–tandem quadrupole mass spectrometer. The analytical conditions were summarized in supplementary material Table S1.

### Data analysis

The analytical method was validated using spiked hair segments according to the method validation guidelines of the Scientific Working Group for Forensic Toxicology (SWGTOX) [25]. The analytes used in this study had already been validated in our previous reports [12, 24, 26]. The validation data were summarized in supplementary material Table S2.

Distribution profiles were generated by plotting drug concentrations per segment (*Y*-coordinate) versus segment numbers from the root to the tip side of the hair strand (*X*-coordinate). Values for peak parameters were measured as follows (see Fig. 1). Segment numbers with the maximal drug concentrations at the root (*C*_R_) and tip (*C*_T_) sides of the hair strands were represented as *S*_R_ and *S*_T_, respectively. The distance between *S*_R_ and *S*_T_ was defined as *D*_R-T_. The intersections at the level of *Y* = *C*_R_/2 with the root curve of the peaks were determined for DP and DDP; twice the distance between an intersection and the line of *X* = *S*_R_ was defined as the width of the root-side peak (*W*_R_). The intersections at the level of *Y* = *C*_T_/2 with the tip curve of the peaks were determined for LD and DLD; twice the distance between an intersection and the line of *X* = *S*_T_ was defined as the width of the tip-side peak (*W*_T_). When the *X*-coordinate of the intersection of a line and a curve was between segments *m* and *m* + 1, the segment number was always numbered as *m* + 0.5, to simplify measurements of peak parameters.

### Effects of washing of hair surfaces

Hair strands that were washed as described above were sonicated with acetone (1.5 mL) for 3 min, followed by dichloromethane (1.5 mL) for 3 min to estimate the effects of vigorously washing the surfaces where the ointment was topically applied. Hair strands were also analyzed as described above.

## Results

### Localization of simultaneously administered drugs in one scalp hair

**Fig. 2:**
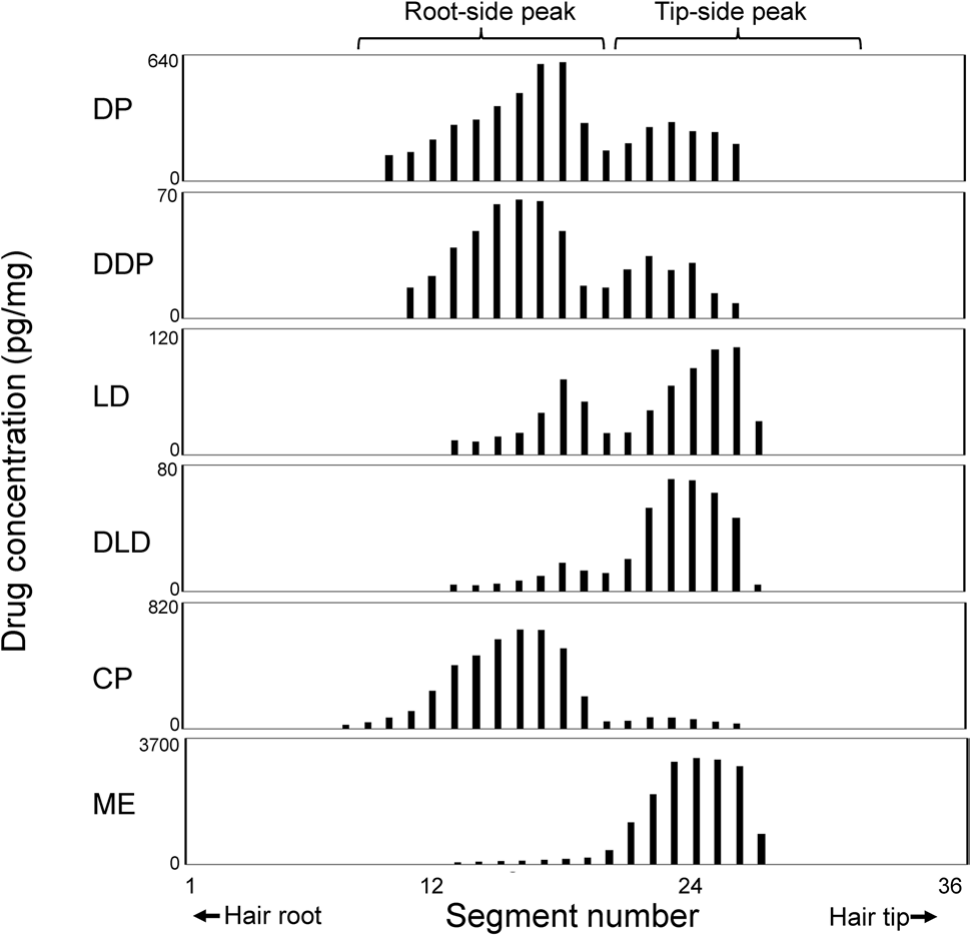
Distribution profiles of analytes in a scalp hair strand collected after simultaneous administration of diphenhydramine (DP), lidocaine (LD), chlorpheniramine (CP), and methylephedrine (ME). Hair ID; APS-31 was collected from the scalp of participant A, who applied the ointment containing DP and LD to the pubic area and orally took CP and ME on the same day. *DDP* desmethyldiphenhydramine, *DLD* desethyllidocaine

Figure 2 shows distribution curves obtained from a scalp hair (hair ID; APS-31) of participant A, who applied the ointment containing DP and LD to the pubic area. The distribution curves of DP and its metabolite, DDP, comprised a major and a minor peak at the root and tip sides of the hair, respectively. In contrast, the distribution curves of LD and its metabolite, DLD, comprised a minor and a major peak at the root and tip sides, respectively. The participant had ingested CP and ME when he applied the ointment to link the peak positions on the distribution curve with the time of drug administration. The positions of the major peaks for CP and ME were near those for DP, DDP, LD, and DLD at the root and tip sides of the hair, respectively.

### Comparison of drug distribution profiles in scalp hair strands according to administration routes

**Fig. 3:**
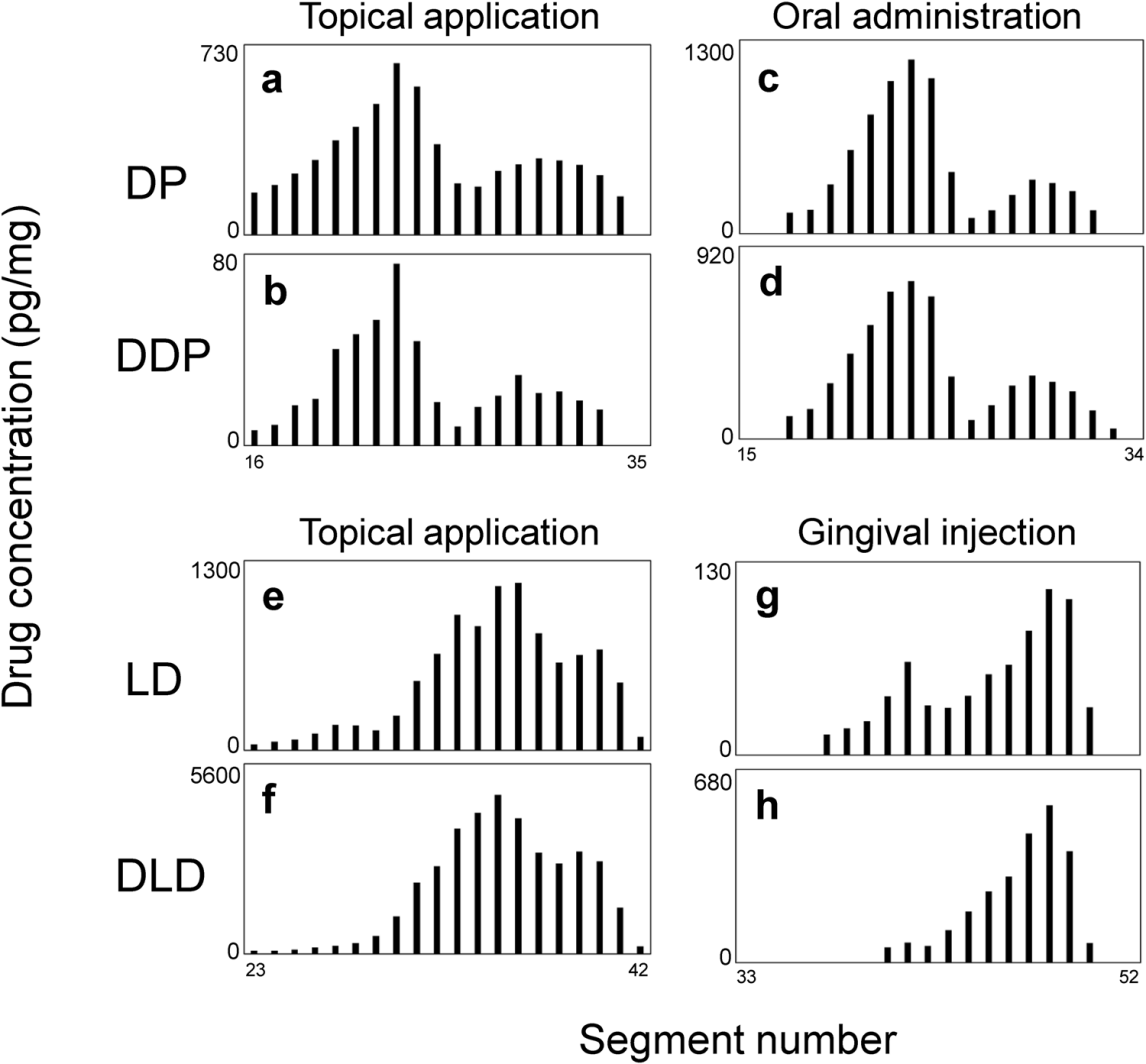
Typical distribution profiles of analytes in scalp hair strands collected after administrating DP and LD via different routes. **a**, **b**, **e**, and **f**: hair (ID; BPS-22) collected from scalp of participant B, who applied ointment containing DP and LD to the pubic area. **c** and **d**: hair (ID; BOS-31) collected from scalp of participant B, who was orally administered DP. **g** and **h**: hair (ID; BIS-53) collected from scalp of participant B, who received gingival injection of LD

We investigated whether drug administration routes could be identified from the profiles of peaks in distribution curves. Both participants orally ingested a single dose of DP. A comparison of the distribution curves between topical and oral administration showed that the profiles of the peak for topical and oral DP and DDP were similar (Fig. 3a–d).

The participants both received a single gingival injection of LD for local anesthesia during dental treatments. We compared distribution profile between LD topically applied to the skin and that gingivally injected, in hairs. Figure 3e–h shows the distribution profiles of LD and DLD with minor root and major tip peaks. The tip-side peaks of LD and DLD were broader for topical application than gingival injection.

To confirm whether peak profiles significantly differed depending on drug administration routes, we analyzed strands of scalp hair from both participants at different sampling times and determined peak parameter values (Tables 1 and 2). The values of *C*_R_ and *C*_T_ for any analyte significantly differed between individual hair strands, regardless of the administration route. Although variations in *W*_R_, *W*_T_, *C*_T_/*C*_R_, and *D*_R-T_ within the same drug administration route tended to be smaller than those of *C*_R_ and *C*_T_, the effects of administration routes did not significantly differ. Therefore, it was difficult to find characteristic peak profiles to identify drug administration routes.

**Table 1:** Peak parameters of diphenhydramine (DP) and desmethyldiphenhydramine (DDP) in distribution profiles in individual scalp hairs collected after the topical and oral administration of DP

	DP^b^					DDP^b^					Metabolite Ratio (%)^c^
Hair ID^a^	*C*_R_	*C*_T_	*C*_T_/*C*_R_	*W*_R_	*D*_R-T_	*C*_R_	*C*_T_	*C*_T_/*C*_R_	*W*_R_	*D*_R-T_	
Topical application											
ALS-11	113	63	0.56	7	7	23	16	0.67	9	7	21
ALS-12	406	214	0.53	9	7	56	22	0.39	7	7	14
ALS-13	372	211	0.57	13	8	54	19	0.36	9	5	15
ALS-21	93	43	0.46	7	6	18	11	0.60	5	10	20
ALS-22	435	172	0.40	11	6	60	9	0.15	5	7	14
APS-31	599	279	0.47	9	5	67	34	0.51	7	6	11
APS-32	334	291	0.87	7	5	29	37	1.28	9	5	9
BPS-11	313	101	0.32	3	7	43	16	0.37	3	6	14
BPS-12	420	228	0.54	7	8	47	14	0.29	3	8	11
BPS-13	526	259	0.49	7	6	47	22	0.48	7	6	9
BPS-21	72	30	0.42	3	7	12	10	0.79	5	6	17
BPS-22	660	273	0.41	7	7	72	28	0.39	7	6	11
Average ± SD	362 ± 192	180 ± 97	0.50 ± 0.14	7.5 ± 2.8	6.6 ± 1.0	44 ± 20	20 ± 9	0.52 ± 0.30	6.3 ± 2.1	6.6 ± 1.4	14 ± 4
Oral administration											
AOS-41	406	79	0.19	5	7	440	127	0.29	5	6	108
AOS-42	279	59	0.21	5	6	323	104	0.32	7	6	116
AOS-51	2534	244	0.10	5	6	1145	166	0.15	5	6	45
BOS-31	1150	360	0.31	5	6	751	304	0.40	7	6	65
BOS-32	1655	431	0.26	5	6	1072	341	0.32	7	6	65
Average ± SD	1205 ± 931	234 ± 165	0.21 ± 0.08	5.0 ± 0.0	6.2 ± 0.4	746 ± 367	208 ± 107	0.30 ± 0.09	6.2 ± 1.1	6.0 ± 0.0	80 ± 31

*ID* identification, *SD* standard deviation

^a^First, second, and third letters of hair ID represent the participant (A or B), drug administration route (O, L, and P, for oral, topical to left axillary, or topical to pubic area, respectively) and scalp hair (S). For example, hair ID: “ALS” refers to a strand of scalp hair collected from participant A after the ointment application to the left axilla. The first and second numbers after capital alphabets represent the serial number of experimental period and the serial number of hair strands at the same sampling time, respectively

^b^Units of peak parameters are pg/mg for *C*_R_ and *C*_T_, and the number of segments for *W*_R_, *W*_T_ and *D*_R-T_. Peak parameters were measured as described in Methods and Fig. 1

^c^Metabolite ratio represents proportion (%) of *C*_R_ for DDP to *C*_R_ for DP

**Table 2:** Peak parameters of lidocaine (LD) and desethyllidocaine (DLD) in distribution profiles in individual scalp hairs collected after topical application and gingival injection of LD

	LD^b^					DLD^b^					Metabolite ratio (%)^c^
Hair ID^a^	*C*_R_	*C*_T_	*C*_T_/*C*_R_	*W*_T_	*D*_R-T_	*C*_R_	*C*_T_	*C*_T_/*C*_R_	*W*_T_	*D*_R-T_	
Topical application											
ALS-11	–^d^	45	–	5	7	–	64	–	5	7	141
ALS-12	29	46	1.6	3	8	11	56	5.1	9	5	122
ALS-13	16	47	2.9	5	7	7	73	10.8	3	6	153
ALS-21	–	20	–	3	8	–	22	–	3	10	113
ALS-22	50	25	0.5	5	6	25	46	1.8	3	6	186
APS-31	61	95	1.6	1	8	14	72	5.2	7	5	76
APS-32	6	70	12.2	5	7	–	64	–	3	8	92
BPS-11	–	622	–	3	–	–	2646	–	3	–	425
BPS-12	91	488	5.4	3	6	–	1919	–	9	–	393
BPS-13	70	682	9.8	3	7	–	3283	–	7	–	482
BPS-21	–	132	–	3	–	–	635	–	11	13	481
BPS-22	160	1174	7.4	9	9	101	4705	46.8	11	12	401
Average ± SD	60 ± 49	287 ± 371	5.2 ± 4.3	4.0 ± 2.0	7.3 ± 0.9	32 ± 39	1132 ± 1614	14.0 ± 18.7	6.2 ± 3.2	8.0 ± 3.0	255 ± 164
Gingival injection											
AIS-51	630	904	1.4	1	9	244	791	3.2	7	6	88
AIS-52	252	418	1.7	1	8	126	517	4.1	7	5	124
BIS-41	153	93	0.6	3	8	120	168	1.4	3	7	181
BIS-42	30	40	1.3	3	8	–	47	–	1	–	117
BIS-51	82	184	2.3	1	8	–	1013	–	3	–	549
BIS-52	77	78	1.0	7	6	130	482	3.7	1	8	614
BIS-53	67	112	1.7	3	7	76	553	7.3	3	7	493
Average ± SD	184 ± 210	262 ± 310	1.4 ± 0.5	2.7 ± 2.1	7.7 ± 1.0	139 ± 62	510 ± 333	3.9 ± 2.1	3.6 ± 2.5	6.6 ± 1.1	309 ± 232

^a,b^See the footnote of Table 1. Second letter (I) of hair ID represents the gingival injection of LD

^c^Metabolite ratio represents proportion (%) of *C*_T_ for DLD to *C*_T_ for LD

^d^*C*_T_/*C*_R_ and *D*_R-T_ could not be measured because root-side peak was undetectable or *C*_R_ was below lower limit of quantification

### Comparison of profiles of analyte distribution among scalp, axillary, and pubic hairs

**Fig. 4:**
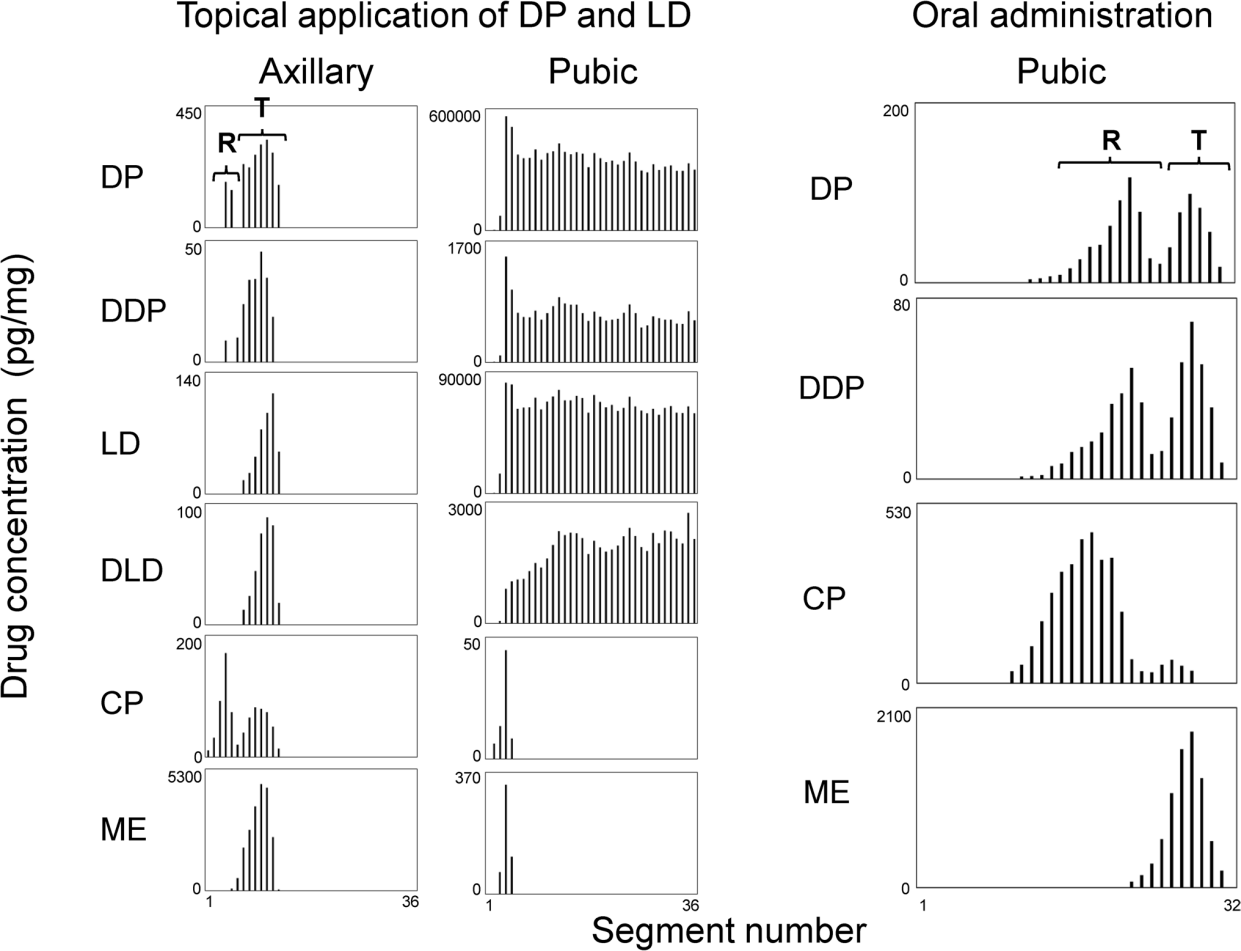
Typical distribution profiles of analytes in left axillary and pubic hair strands. In topical application, the strands of left axillary and pubic hair were collected 14 days after participant A applied the ointment containing DP and LD to the pubic area and orally ingested CP and ME on the same day. In oral administration, the pubic hair strand was collected 15 days after participant A orally ingested DP. CP and ME were orally ingested on the day following DP ingestion. **R** and **T** represent the root-side and tip-side peaks, respectively

Figure 4 shows typical distribution profiles of analytes in strands of axillary and pubic hairs obtained from participant A. Strands of axillary and pubic hairs were collected 2 weeks after the participant applied the ointment containing DP and LD to the pubic area. The participant had orally ingested CP and ME on the same day as the ointment application. Drug peaks were detectable in only 2 of 6 axillary hair strands analyzed. The positional relation between analyte peaks in axillary hair was similar to that in scalp hair. Whereas, high concentrations of DP, LD, and their metabolites were detected along the shafts of pubic hairs growing in the area where the ointment was applied.

Additionally, strands of axillary and pubic hairs were collected from participant A 2 weeks after DP was orally ingested. The participant also orally ingested CP and ME on the day following DP ingestion. Drug peaks were detectable in only 1 pubic hair strand out of 3 axillary and 3 pubic hair strands analyzed. The positional relation between analyte peaks in the pubic hair was also similar to that in scalp hair (see Fig. 4, right panel; Fig. 3c, d).

### Effect of washing hair surfaces contaminated with drugs

**Fig. 5:**
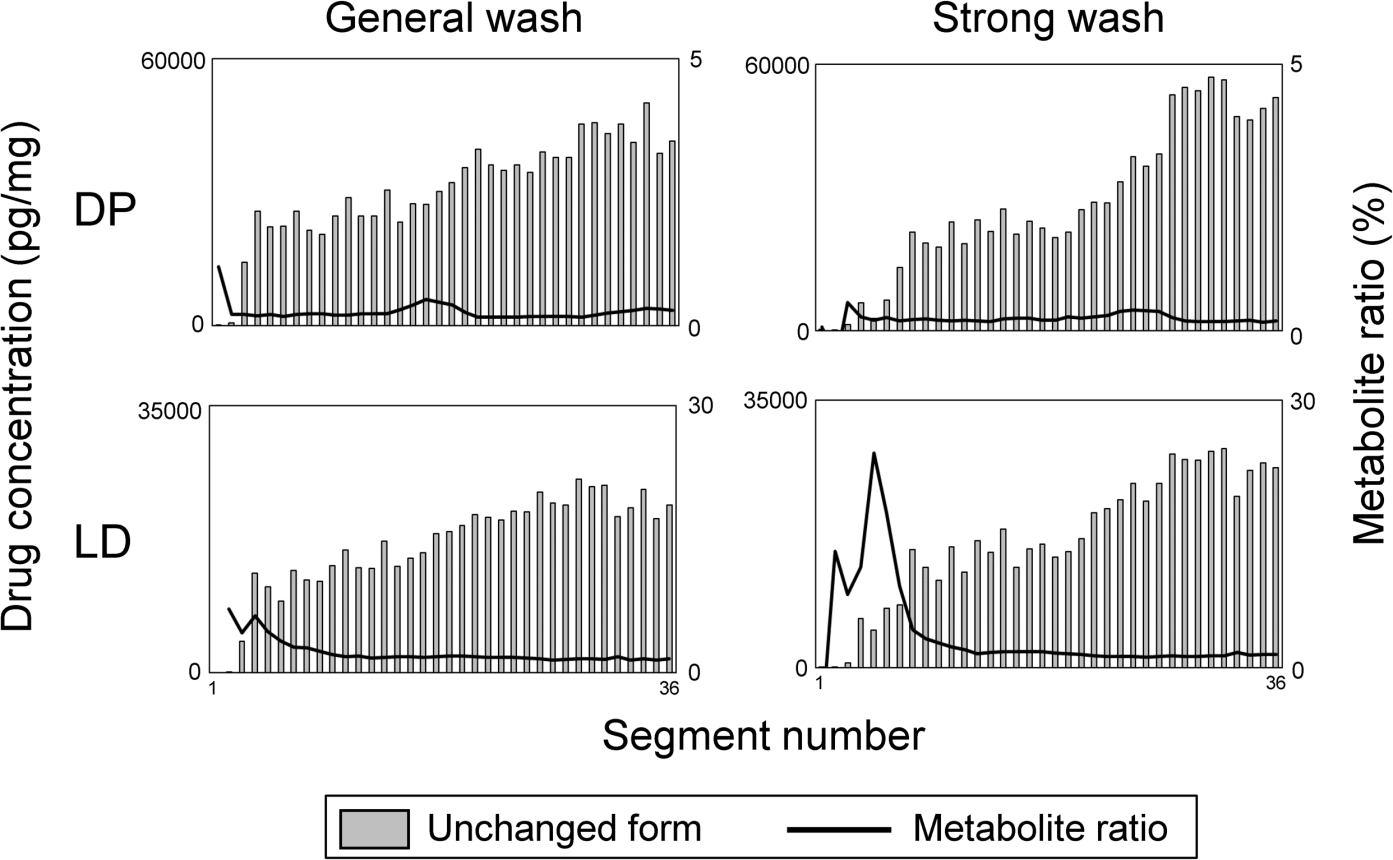
Typical distribution profiles of analytes in pubic hair strands in the area where ointment was applied. Strands of pubic hair collected from participant B 14 days after application of ointment containing DP and LD to pubic area were generally washed and then sonicated in acetone and dichloromethane. Metabolite ratio: proportion (%) of DDP to DP or DLD to LD concentrations in each hair segment

High concentrations of analytes were detected in hairs growing in the axillary and pubic areas where the ointment was applied. We washed the hair surfaces with aqueous SDS, water, and methanol, then sonicated them in acetone and dichloromethane for 3 min each. The analyte concentrations in these hairs did not significantly decrease (Fig. 5).

Concentration ratios of metabolites to unchanged DP and LD in each pubic hair segment were measured along individual hair strands. The ratios for both DP and LD were essentially constant at < 0.5% and < 3%, respectively, along the hair shaft, at the tip from segment 10 (4 mm from the hair root end). In contrast, ratios of metabolites fluctuated near the roots of the hairs, especially for those of LD (Fig. 5).

## Discussion

### Routes of uptake into scalp hair differ depending on administered drugs

We previously showed that CP and ME are located at different positions in strands of scalp hair even when simultaneously ingested [12]. This is because CP is predominantly incorporated into the hair follicle via the bloodstream, whereas ME is predominantly incorporated into hair via the sweat and sebum secreted inside/near the scalp. The uptake routes of DP and LD into scalp hair were the same as those of CP and ME, respectively. The relationship between the uptake route and chemical properties of drugs has not yet been elucidated, although micro-segmental analysis has shown that many drugs were incorporated mainly via the bloodstream at hair follicles [12, 24, 27, 28].

### Identification of administration routes of drugs

Variations in the C_R_ were large even when drugs were administered via the same routes (Tables 1 and 2). Peaks for LD and DLD were not found at the root sides of some hair strands, because these peaks were small and obscured at the bottom of the tip-side peaks (Table 2, Figs. 2 and 3). This could be because the amount of drug incorporated into hair follicles depends on the activity of individual hair matrices. Additionally, the magnitude of the relationship between C_R_ and C_T_ was reversed in three hair strands [APS-32 for DDP (Table 1), ALS-22 and BIS-41 for LD (Table 2)]. Amounts of sweat and sebum secreted onto the scalp vary among individuals, as well as time, and the scalp region, and might affect the C_T_. Characteristic peak profiles on distribution curves are more important to identify drug administration routes than drug concentrations. Although some peak profiles differed (Fig. 3e–h), all peak parameters considerably varied among individual hair strands even when drugs were administered via the same route. Therefore, peak profiles did not significantly differ according to drug administration routes in the present study. However, the other drugs that can be administered via different routes, such as inhalation and intravenous injection, should also be investigated. If the peak profile of a specific drug significantly changes depending on the administration route, the distribution profiles obtained by micro-segmental analysis would be useful to estimate which route had been applied in drug-related crimes.

### Effectiveness of alternative hairs to scalp hair in drug testing

Axillary and pubic hairs can be used as alternatives when scalp hair is not available for drug tests due to alopecia or heads shaved to hide evidence of drug use. We first applied micro-segmental analysis to axillary and pubic hairs. The distribution profiles in most axillary and pubic hairs, which are located far from areas where the ointment was applied, did not contain any significant analyte peaks. In contrast, the distribution profiles for all plucked scalp hair strands showed drug peaks in specific regions according to the time when the ointment was applied to the pubic area (Fig. 2). The ratios of axillary and pubic hairs in the catagen and telogen phases were higher than that in scalp hair (40–60% vs. ~ 15% [29]). In fact, only 2 of 9 axillary hair strands and only 1 of 3 pubic hair strands analyzed had sufficient amounts of analytes, regardless of the administration routes (Fig. 4). Thus, the risk of selecting individual hair strands in the catagen/telogen phases is higher among those obtained from the axillary and pubic areas than from the scalp. Therefore, individual axillary and pubic hairs are not effective as the alternatives to single scalp hairs for drug tests from the perspective of hair matrix activities and hair cycles. Although conventional hair analysis has detected drugs from axillary and pubic hairs [19–22], tens of strands (at least 10 mg) are usually combined into one analytical sample. If micro-segmental analysis of axillary or pubic hairs is required under real circumstances, more hair strands must be analyzed to obtain the distribution profiles of hair strands in the anagen phase with high reproducibility.

### Distinguishing external contamination from drug administration with hairs

High concentrations of DP and LD were detected along the shafts of all analyzed hair strands in the area where the ointment was applied because of direct contact with the pubic hair shaft (Fig. 5). However, DP and LD were essentially undetectable near the hair root because it was below the epidermis, or was generated de novo after ointment application. We confirmed that drugs adsorbed onto the hair surface was difficult to remove by harsh treatments such as sonication in various solvents, once hair strands were contaminated by the procedures with large amounts of drugs such as ointment application.

The respective DDP and DLD metabolites of DP and LD were also detected along the shaft of all analyzed hairs in the area where ointment was applied. This was attributed to decomposition of the unchanged form in air. In fact, DDP and DLD were detected in extracts of the ointment that had been exposed to air at 22 °C for 24 h (unpublished observation). Therefore, the presence of metabolites cannot necessarily be a marker to distinguish external contamination from drug administration by hair analysis when the metabolites and the decomposed compounds are the same.

The concentrations of DP and LD detected at the shafts of hairs growing in the area where ointment was applied (Fig. 5) exceeded the maximal concentrations in scalp hair (Tables 1 and 2). Additionally, the metabolite ratios at the hair shaft in the contaminated hair were lower than those at the maximal concentrations in scalp hair. The concentration of the unchanged form, the metabolite ratio, and the hair regions where the unchanged form and metabolites were detected would be important to distinguish external contamination from drug administration.

The slight increase in the metabolite ratio near the hair root in Fig. 5 implies that the detected analytes were generated by uptake via the bloodstream, sweat, and sebum, and not by external contamination. To precisely identify such characteristics on drug distribution profile, it is impossible to use the conventional hair analysis, in which hair surface contamination is evaluated only by comparing the amounts of a drug in solvents used to wash the hairs with that of the extracted drug [30–32]. Differences in the amounts of drugs between wash solutions and extracts can be insufficient to evaluate contamination on hair surfaces, because the amounts of drugs in individual hair strands where drugs are localized at specific hair regions are averaged in the conventional hair analysis. Micro-segmental analysis can clearly differentiate distribution profiles between external contamination and drug administration (Figs. 1, 2, 3, 4, 5). For instance, whether a detected drug was derived from external contaminants can be determined if concentrations of an unchanged drug remain high and metabolite ratios remain low along the hair shaft (Fig. 5). In contrast, if the distribution profile has narrow peaks in specific regions, the detected drug would be regarded as being administered, because the partial adsorption of drugs onto the hair surface is difficult to create a distribution curve with major and minor peaks artificially (Figs. 2, 3, 4). Distribution profiles obtained using micro-segmental hair analysis would be useful not only to estimate when a drug was administered, as we previously reported [24, 27, 28], but also to identify whether the hair had been in contact with drugs externally.

## Conclusions

This study revealed the following points: the distributions of DP and LD differed in individual scalp hair strands; administration routes of DP and LD could not be confirmed based on peak profiles in distribution curves; axillary and pubic hairs were not effective alternatives to scalp hair in single hair analysis; and detailed visualization of drug distribution profile in hair using the micro-segmental analysis clearly distinguished drug administration from external drug adsorption (contamination). This analytical method would be useful to elucidate how drugs are incorporated into hair and to estimate the circumstances of drug use in drug-related crimes.

## Supplementary Information

Below is the link to the electronic supplementary material.

Supplementary file1 (PDF 62 kb)
