# Supplementary material for: Distribution profiles of diphenhydramine and lidocaine in scalp, axillary, and pubic hairs measured by micro-segmental hair analysis: good indicator for discrimination between administration and external contamination of the drugs
Source: Forensic Toxicol. 2021 Jul 31;40(1):64–74. doi: 10.1007/s11419-021-00590-x (PMC9715455; doi:10.1007/s11419-021-00590-x)
Supplement: Supplementary file 1 — Supplementary file1 (PDF 62 kb) [file 11419_2021_590_MOESM1_ESM.pdf]

Supplementary material

Table S1 Analytical conditions for the detection of analytes in hair samples

LC–MS/MS	Waters ACQUITY UPLC I-Class and Xevo TQ-S
Column	Imtakt Cadenza CD-C18 HT (150 mm × 2.0 mm, 3 μm)
Column temp.	40 °C
Flow rate	0.2 mL/min (0–0.2 min) – 0.3 mL/min (0.2–2 min) – 0.4 mL/min (2–4 min) – 0.5 mL/min (4–5 min)
Mobile phase	A: 5 mM ammonium acetate + 0.05 % formic acid<br>B: Acetonitrile<br>5 % B (0–0.2 min) – 20 % B (0.2–2 min) – 50 % B (2–3 min) – 95 % B (3–4.5 min) – 5 % B (4.5–5 min)
Ionization	Electrospray ionization
Acquisition mode	SRM
SRM conditions	

Analyte	Monitoring ion (m/z)	Cone voltage (V)	Collision energy (eV)
DP	256 > 167	5	15
DDP	242 > 167	5	15
LD	235 > 86	30	15
DLD	207 > 58	4	12
CP	275 > 230	2	16
ME	180 > 162	4	12
CP-d_6	281 > 230	2	16

Liquid chromatograph-tandem mass spectrometer (LC–MS/MS), selected reaction monitoring (SRM), diphenhydramine (DP), desmethyldiphenhydramine (DDP), lidocaine (LD), desethyllidocaine (DLD), chlorpheniramine (CP), methylephedrine (ME)

Table S2 Analytical validation using spiked 0.4-mm hair segment samples

Analyte	LOD (pg/mg)	LLOQ (pg/mg)	Spiked conc. (pg/mg)	Ac (% bias)[a]	Pc (% CV)[b]	MF (%)[c]	Recovery (%)[c]
DP	5	50	50	−7.4	14.0	88 ± 12	76 ± 11
			1000	1.6	1.9	120 ± 6	100 ± 3
DDP	5	25	50	−11.0	5.7	85 ± 17	88 ± 13
			1000	2.2	3.5	124 ± 10	102 ± 5

LD	5	50	50	−4.8	9.5	103 ± 15	96 ± 4
			1000	−0.3	4.2	114 ± 5	115 ± 7
DLD	5	25	50	−3.5	7.5	117 ± 8	112 ± 8
			1000	1.4	7.1	120 ± 7	123 ± 8

Limit of detection (LOD), lower limit of quantification (LLOQ), accuracy (Ac), precision (Pc), coefficient of variation (CV), matrix factor (MF)

The weights of each 0.4-mm blank hair segment were regarded as 4 μg/segment

[a] Average of five measurements

[b] Calculated based on measurements on five different days

[c] Calculated using 0.4-mm hair segments from six drug-free subjects
